# Social sciences research in neglected tropical diseases 2: A bibliographic analysis

**DOI:** 10.1186/1478-4505-9-1

**Published:** 2011-01-06

**Authors:** Daniel D Reidpath, Pascale Allotey, Subhash Pokhrel

**Affiliations:** 1 Global Public Health, Jeffrey Cheah School of Medicine and Health Sciences, Monash University, Malaysia; 2 Health Economics Research Group, Brunel University, West London, UK

## Abstract

**Background:**

There are strong arguments for social science and interdisciplinary research in the neglected tropical diseases. These diseases represent a rich and dynamic interplay between vector, host, and pathogen which occurs within social, physical and biological contexts. The overwhelming sense, however, is that neglected tropical diseases research is a biomedical endeavour largely excluding the social sciences. The purpose of this review is to provide a baseline for discussing the quantum and nature of the science that is being conducted, and the extent to which the social sciences are a part of that.

**Methods:**

A bibliographic analysis was conducted of neglected tropical diseases related research papers published over the past 10 years in biomedical and social sciences. The analysis had textual and bibliometric facets, and focussed on chikungunya, dengue, visceral leishmaniasis, and onchocerciasis.

**Results:**

There is substantial variation in the number of publications associated with each disease. The proportion of the research that is social science based appears remarkably consistent (<4%). A textual analysis, however, reveals a degree of misclassification by the abstracting service where a surprising proportion of the "social sciences" research was pure clinical research. Much of the social sciences research also tends to be "hand maiden" research focused on the implementation of biomedical solutions.

**Conclusion:**

There is little evidence that scientists pay any attention to the complex social, cultural, biological, and environmental dynamic involved in human pathogenesis. There is little investigator driven social science and a poor presence of interdisciplinary science. The research needs more sophisticated funders and priority setters who are not beguiled by uncritical biomedical promises.

## Introduction

I have never read a research proposal written by a social scientist that was worth funding.^1^

Human pathogens have adapted to take advantage of the behaviour and social and nature of their human hosts, including adaptations to take advantage of the structured nature of the societies in which humans live [1,2]. The survival of a subset of those pathogens, the ones that are vector borne, relies on the pathogens concomitant adaptation to the vector-arthropods that have themselves adapted (and continue to adapt) to take advantage of the behaviour and social nature of humans, including the structured nature of the societies in which humans live [3-5]. Vector borne diseases represent a rich and dynamic interplay between the vector, the host, and the pathogen; but it is an interaction that occurs within a social and cultural context as much as it is one that occurs within a physical and biological context.

Critically, aspects of pathogenesis as diverse, and prima facie non-social, as the jump of a pathogen from one host species to another [6,7], or the development of drug resistance [3,8] have significant social components. As an example, consider drug resistance, particularly arising from the counterfeiting of drugs [9,10]. Counterfeiting is an entirely social phenomenon driven by healthy profits margins. Counterfeit antimalarial drugs which contain sub-clinical doses of artesunate have been identified, and their presence has plausibly driven drug resistance [11,12]. Drug resistance, however, does not only occur because of the malign intentions of criminal elements, and can also be driven by other social and behavioural phenomenon such as poor prescribing practices of medical practitioners and pharmacists, or poor compliance by patients [13,14]. Drug resistance arises, therefore, out of a social and cultural context within which drug treatment is delivered and managed, and the biological context of the hosts' and agents' response to the drug delivery regime.

Under this scenario of complex interactions between social and biological forces, one might imagine that the social sciences would play a significant, if not central role, in the development of evidence related to understanding and managing pathogenesis in general, and in the management of the arthropod borne, neglected tropical diseases (NTDs) in particular.

As an entry point for thinking more concretely about where the social sciences contribution to NTDs research may lie, consider the kinds of litter that humans generate, and the manner in which they manage that litter. The creation and management of litter has a social, cultural, and behavioural basis related to (but not limited to) our diets and shopping habits, the kinds of packaging that industries use, our housing, our cultural views and practices related to the management of waste (and even the definition of one thing as waste and another as a possession), the local municipal infrastructure for the collection and management of waste, government policies, and physical isolation.

For *Aedes albopictus*, one of the mosquito vectors of the dengue virus, the generation and management of litter around the home will have a direct impact on whether there is an abundance of breeding sites conveniently close to human blood meals, or whether the breeding sites are sparse [15]. Furthermore, and in keeping with the dynamic nature of the relationship between the vector and the host, variations in social structure, cultural practice, or individual behaviour, will also affect the need for the mosquito to adapt its breeding habits in response to changes in the nature and quantity of the human litter [15,16].

Some of the social and behavioural aspects associated with pathogenesis may, thus, be quite micro-level in their scope. These would include household factors associated with pathogenesis such as physical location, the civil engineering of the housing [17], demographic profile, and individual knowledge and behaviour [18-21]. Micro-level factors will be more readily influenced by individual and household level activities. Other macro-level factors associated with pathogenesis will include levels of poverty in the region, the level of development, the town planning codes and practices [22], the sanitation services infrastructure, the political and economic capacity (or desire) to restructure those things, and the cultural views and practices of the community.

Once one understands that the relationship in the host, agent (vector), and environment "triad" embeds inescapable social dimensions, the nature and scope of those social dimensions become available for systematic investigation as a part of a larger NTDs research agenda. One could, by way of examples, investigate social and behavioural aspects that affect: (i) the pre-existing risk of pathogenesis, (ii) the prevention of pathogenesis, and (iii) the treatment and management of pathogenesis and the morbidities arising from it. At a micro-level one could examine, for instance, the presence of social risk factors and the distribution of a particular known social risk factor in the population, or the local understanding of a disease so that more appropriate management strategies could be developed [23-25]. At a macro-level one could, for instance, investigate the political economy of disease and how it effects health resource priorities in government and their impact on one or more NTDs. One could also look at the interrelationship between arms of government such as Agriculture, Education, Health, Finance, Transportation, and Communication and consider the effects that these individually or in aggregate have on the spread or control of disease.

A research agenda with this kind of scope would have a considerable amount to offer in the understanding, control, and management of NTDs. It would also carry the risks of any activity carried out in a silo: when one has a hammer, everything looks like a nail. Understanding and managing human pathogenesis may have inescapably social dimensions, but it is equally bound to biological and clinical dimensions. The potential of inter- and multi-disciplinary research^2^, therefore, combining social and biological facets (e.g., social factors affecting the physical and economic distribution of onchocercal drug resistance in the population), social and clinical questions (e.g., social factors affecting the sustained implementation of a treatment strategy in a community), or indeed social questions with questions from other entirely different disciplines such as architecture or engineering would be significant (e.g., social dimensions of housing design and infection rates [17]).

This kind of call for interdisciplinary research, particularly with social sciences inclusion, is not new [26]. Individual researchers, and organizations working in the NTDs and infectious disease more generally, have argued explicitly for the value of social sciences research [27-29], or have used social science arguments to advance the political cause of NTDs research^3^. At the same time, however, researchers have argued that too much weight is being given to the social sciences, and more weight needs to be given to other disciplines, particularly the laboratory based sciences [30]. Against this backdrop of disciplinary demarcation disputes in what is clearly a multidisciplinary field, there are likely to be three problems for social sciences research:

1. Research will remain largely within disciplinary silos;

2. Biomedical research will dominate; and

3. Where efforts are made to encourage more than one discipline engaging with a problem, the work will be *multi*disciplinary rather than *inter*disciplinary:

• The contributions from different disciplines will be treated hierarchically, and

• The social sciences research will be responsive rather than investigator driven.

As a consequence of the last of these sub-points one would anticipate that the social science research will be conducted under the mantle of disease/cure focussed, biomedical research. This would arise if the role of the social research is as an adjuvant activity to support the curative activities of biomedical research. For an example, see Parker and Allen [31] in this series. Under these conditions the research will often reduce to a "cookbook" style of evaluation in support of some programmatic intervention. For examples, see Pokhrel et al. in this series [32]. One might anticipate that if this were the research being conducted, it would have a limited or peripheral impact on understanding or managing of NTDs.

In this paper, we examine the contribution of different disciplines to NTDs research in the published literature focusing on the role of the social sciences. The purpose of the review is to provide a baseline for discussing the quantum and nature of the science that is being conducted.

## Method

A bibliographic analysis was conducted of NTDs related research papers published over the past 10 years in (a) biomedical sciences (i.e., medicine and the human health sciences), and in (b) the social sciences. The analysis had textual and bibliometric facets. Our reason for looking at only published papers is straight forward. Unpublished research does not have the breadth of impact that is achieved by published research. It is often not peer reviewed, and is not open to the same scrutiny and discussion often required to influence policy.

### Definitions

Defining, or operationalising "the social sciences" is key to determining the quantum and nature of the contribution they make to NTDs research. Unfortunately, what defines a social science is itself perennially contested [33], and methodological and disciplinary debates about what is or is not a science, a social science, or whether social science is even a science, are commonplace (see for instance [34]). Colleagues, particularly in disciplines such as psychology and economics - in which the focus can be (but often is not) on the individual - have at times been quick to distance themselves from the social sciences. Although it is not uncommon for researchers from these disciplines to take up positions or research funding designated for social scientists.

The matter is further complicated, because the boundaries between the social sciences and biomedicine are not fixed, with many clinicians, identifying strongly with Rudolph Virchow's famous view that medicine is a social science [35]:

Die Medizin ist eine soziale Wissenschaft, und die Politik ist nichts weiter als Medizin im Großen.

Medicine is a social science, and politics is nothing more than medicine writ large.

Some even hold the disturbingly arrogant position that medicine is, in fact,"the most scientific of social sciences" (p.55) [36].

For our purposes, we operationalised the social sciences according to the database abstracting services definition, to include the key areas of arts, business, economics, psychology, and decision science. The Medical and human health sciences were similarly operationalised according to the abstracting services approach. The definition, is thus, instrumental in nature. An article belongs, *prima facie*, to the social sciences if, in conducting a search using the social science disciplinary categories provided by the database abstracting service, that article is returned. This means that medicine and the human health sciences may be social sciences, but only when the work for medicine and human health sciences is abstracted simultaneously under the social sciences.

The reasonableness of this approach is discussed later, and caveats occur in the analysis of the Results.

### The Diseases

The aim of this review was not the comprehensive coverage of the social sciences in all NTDs research, but to examine a handful of examplar diseases. The choice of the diseases was somewhat arbitrary settled on by mutual agreement of the authors. Four arbor NTDs were the focus of the present analysis: chikungunya [37], dengue [38], visceral leishmaniasis [39], and onchocerciasis [40] - see Table 1. Three of the diseases were of direct interest to the original funders of this review - dengue, visceral leishmaniasis and onchocerciasis (see Acknowledgements), and one of the diseases (chikungunya) was selected because of the recent increase in profile that it appeared to have achieved.

**Table 1:** The disease, agent vector, and general geographical distribution of the four reviewed diseases.

Disease	Agent	Vector	Distribution
Chikungunya	Virus	Mosquito species: *Aedes aegypti/Aedes albopictus*	Asia, Africa, islands of the Indian Ocean
Dengue	Virus (genus *Flavivirus)*.	Mosquito species: *Aedes aegypti/Aedes albopictus*	South Asia and South East Asia, the Pacific and South and Central America
Visceral Leishmaniasis	Protozoan (various species of the genus *Leishmania)*.	Sandfly: Various species of sandfly (genus *Lutzomyia *in the New World, and *Phlebotomus *in the Old World).	South America, East Africa, Mediterranean, South Asia, China
Onchocerciasis	Microfilarial nematode (Species: *O. volvulus)*.	Blackflies of the genus *Simulium*	99% of cases occur in Africa, mainly in the central belt of Africa from the West to East coast

### Data

#### Source

The data for the bibliometric and textual analyses were obtained from Scopus™, an on-line, commercial abstract and citation database service offered by the publishing house, Elsevier. According to Elsevier, Scopus is the largest abstracts and citation database in the world. "All titles that conform to academic quality norms, specifically peer-review, and are published in a timely manner are accepted for consideration [in the Scopus database]" [41]. Significantly, the Scopus database also includes all articles abstracted in the US National Library of Medicine's Medline, PubMed databases, and it abstracts 350 book series. The lack of books in the database may bias the findings, particularly in the social sciences which historically have favoured books and book chapters. The bias is likely to be less than in the past, however, with an increasing trend in the social sciences towards the publication of research in journals [42].

#### Extraction

Articles relating to the four NTDs were identified by searching against the title, abstract and key words fields (i.e., TITLE-ABS-KEY). for "chikungunya", "dengue", "visceral leishmaniasis", and "onchocerciasis," respectively. It was possible to limit searches further to those articles that fell within particular disciplines, or subject areas. For the social sciences articles, searches were limited to the Scopus subject areas of: arts, busi[ness], dec[sion], econ[omics], psyc[hology], and soci[al] - this conforms with the Scopus definition of social sciences. For searches against the biomedical literature, searches were limited to the Scopus subject areas of: medi[cine], nurs[ing], and heal[th]. Veterinary science, for instance was excluded from the search, because the focus was human health. Only documents of type "ar[ticle]" were selected, to focus on research articles rather that reviews - although the separation between these activities is certainly open to debate, and the database itself is not consistent on this. The searches were also limited to those papers published in the last 10 years (i.e., published after 1999)..

By way of an example, the search in the social sciences for onchocerciasis literature was:

TITLE-ABS-KEY (onchocerciasis). AND DOCTYPE (ar). AND SUBJAREA (arts OR busi OR deci OR econ OR psyc OR soci). AND PUBYEAR AFT 1999 and the equivalent search in the biomedical literature was:

TITLE-ABS-KEY (onchocerciasis). AND DOCTYPE (ar). AND SUBJAREA (medi OR nurs OR heal). AND PUBYEAR AFT 1999

After the relevant articles were identified, selected fields including a unique identifier for each article, the year of publication, the number of citations, the article title, the journal title, the authors, the abstract, and the authors' affiliations were downloaded.

#### Analysis

The analysis combined exploratory and descriptive techniques for the available quantitative data, and textual analysis for the published abstracts The data from each search was imported into its own table in a relational database. Using the unique article identifier as the primary key it was possible to examine the degree of overlap between the biomedical and social sciences searches for each disease.

The research was conducted entirely on Open Source platforms [43]. The operating system was the GNU/Linux based Ubuntu 9.10 [44]. Word processing and database management was handled in OpenOffice [45] using the Zotero bibliographic database [46]. The data analysis was handled in the R statistical package [47].

## Results

Of the four NTDs investigated in this paper, over the past 10 years dengue had the most publications (n = 2,344), followed by visceral leishmaniasis (n = 1,648), onchocerciasis (n = 483), and chikungunya (n = 274). The relative lack of publications associated with chikungunya prompted an investigation of earlier citations. Scopus records a total of 774 articles associated with chikungunya, the first of which was published in 1957 [48]. Scientific interest in the disease, as demonstrated by publications, remained at low levels, rising in the late 1960's but declining again by the early 1980's. It was not until 2006 that there was a resurgence in scientific publications in the area. In contrast the annual number of dengue publications has increased steadily from the 1960's, a picture that is somewhat similar to that of visceral leishmaniasis and onchocerciasis - although the latter has never attracted quite the same level of interest as dengue or visceral leishmaniasis.

The quantum of disease specific research conducted in the biomedical sciences and social sciences is shown in Table 2. For all four diseases, the biomedical sciences, unshared with the social sciences, account for more than 95% of the publications, and in the case of visceral leishmaniasis, it accounts for more than 99% of the publications. Conversely, the social sciences, unshared with the biomedical sciences, usually accounts for less than 1.5% of the publications, and in one case around 0.1%. Joint biomedical and social sciences publications are similarly sparse. In aggregate, as a percentage of the published research related to chikungunya, dengue, onchocerciasis and visceral leishmaniasis, the research identified as belonging to the social sciences represents about 2.1% of the total output.

**Table 2:** For each disease, the number of publications is a discipline and disease area is shown.

Disease	Biomedical Science	Joint	Social Science	Total
Chikungunya	267 (97.4)	4 (1.5)	3 (1.1)	274
Dengue	2,270 (96.8)	33 (1.4)	41 (1.7)	2,344
Visceral Leishmaniasis	1,641 (99.6)	5 (0.3)	2 (0.1)	1,648
Onchocerciasis	469 (97.1)	8 (1.7)	6 (1.2)	483

The percentage of the total number of publication for a disease is shown in parentheses.

The Scopus abstracting service further divides the biomedical literature into component disciplines, and within this there was overlap. Thus, almost all the papers were indexed as medicine, and some were associated with other disciplines as well. Immunology and microbiology accounted for around 50% of the biomedical literature for all diseases except chikungunya (34%). Biochemistry, genetics and molecular biology accounted for between 3% (onchocerciasis) and 9% (visceral leishmaniasis) of the biomedical literature. This analysis by component disciplines makes less sense for the social sciences literature, because those disciplines were so poorly represented; and allocating one or two papers to this discipline or that discipline, is hardly informative.

Of more interest in this case is to turn to the abstracted data themselves to obtain a sense of the kind of social science research that was being conducted in these NTDs. The social sciences articles and the joint social sciences and biomedical articles are discussed as a whole.

There were few social sciences articles in the chikungunya, visceral leishmaniasis, or onchocerciasis literature over the past 10 year, so the presentation of data is in the form of a series of cases. Nonetheless, there are pertinent themes that emerge.

A cursory examination of the institutional affiliation of the authors reveals an interesting phenomenon. Departments of zoology, virology, molecular biology, microbiology, etc., that is, departments associated with strong clinical and biomedical disciplines, are producing a surprising number of the social sciences papers. In contrast, there are no departments of sociology or psychology producing biomedical papers. One of two possibilities come to mind. The first, and most positive interpretation, is that biomedical departments are broad-based inter- or multi- disciplinary centres of NTDs research. The second, and less benign interpretation is that "social science research" has become a cookbook activity in the NTDs area, with all the attendant dangers of thoughtless research. For a discussion of some of these issues, see for instance [49-51].

### Sensitivity analysis

The search strategy itself could miss studies that others might consider to be social science research, because of limitations in the database abstraction process. By way of a sensitivity analysis, this was checked for the disease onchocerciasis by removing any disciplinary restriction and using the more inclusive search term:

TITLE-ABS-KEY (onchocerciasis). AND DOCTYPE (ar). AND PUBYEAR AFT 1999,

The titles of all the extracted papers were examined, and a subset were identified for which the abstracts were also read. Studies judged by the authors (all of whom are social scientists from three distinct disciplines) to have a social sciences component were then compared with the papers identified using the Scopus disciplinary identifiers.

Onchocerciasis was used as a test of the comprehensiveness of the Scopus search engine to identify social science research. When the search was conducted without any disciplinary restriction, 701 articles were identified. Of these 701 articles, 24 were social science articles that had not been identified in the original search using the Scopus disciplinary restrictions. This represents a substantial increase over the 14 originally identified articles, but only brings the total number of social science articles up to 38, or 7.5% of the 507 biomedical and social sciences articles. The 24 newly identified studies were almost exclusively joint social science and biomedicine publications, and the majority (n = 14) related to implementation research, such as studies of particular strategies for the community directed delivery of ivermectin or doxycycline [52-56].

### Chikungunya

Three of the chikungunya papers could be characterised as socioepidemiological. Each one focused on a particular outbreak: one each from the the islands of Mayotte and Reunion in the Indian Ocean [57,58], and a third described an outbreak in Italy [59]. The Mayotte and Reunion papers were multidisciplinary looking at clinical, behavioural, attitudinal, and environmental factors. Two papers were discussion pieces on the globalisation of infectious diseases [60,61]; and one paper was a textual analysis of the political discourse of risk that occurred in Metropolitan France as it managed the outbreak on Reunion [37]. This latter paper is the only one that could be described as an empirical, macro-level study, and stands out for this as unusual across the four NTDs areas examined.

### Visceral Leishmaniasis

The visceral leishmaniasis literature is quite different, with most of the studies barely (or not at all) characterisable as coming out of the social sciences. Three of the "social science" studies described n-of-1 clinical case studies [62-64]. One "social science" study was of asymptomatic individuals who were antibody positive for *T. Cruzi*, and the serological complications associated with those who were infected with visceral leishmaniasis [65]; one study was of the distribution of the sand-fly around US military bases in North America (pre-empting the accidental importation of visceral leishmaniasis by troops returning from Iraq) [66] There was a case-control study of environmental risks associated with having visceral leishmaniasis [67], and another study looking at the distribution of canine and human visceral leishmaniasis in Venezuala [68] These latter two studies have broadly social components, but remain largely biomedical in nature.

### Onchocerciasis

The social sciences literature relating to onchocerciasisis is larger (i.e., twice as many papers as the chikungunya or the visceral leishmaniasis literature) and more focussed. Speculatively, this may be attributable to the presence of the unifying onchocerciasis control programmes in Africa [69]. Furthermore a significant proportion of this literature was produced by TDR supported scientists as part of a concerted effort to increase the engagement of social science in disease control programmes [29]. A control programme provides a clear line of research, for instance, around implementation and programmatic evaluation [26]. Two of the studies (one qualitative and one qualitative and quantitative) examined community directed treatment programs [70,71], one study looked at social inequity in treatment seeking [72], and another study looked at the implications of the gender of the community workers on the delivery of treatment [73]. There was research from a large multi-country study looking at gender differences associated with the stigma of onchocercal skin disease [74]; and a qualitative study examined the community perceived benefits of ivermectin use in the treatment of onchocerciasis revealed an under-estimation of the district's ivermectin needs [54]. It should also be noted that most of the studies have been poorly cited, with an average of 2.5 citations for each of the implementation research studies.

One study was misclassified as onchocerciasis research, and a few studies had too little information to evaluate. There was also a paper discussing tensions between the need to preserve wetlands, and the potential health hazards posed by arbor infectious diseases, including onchocerciasis [75].

One research note that stood out for its potential described a "multidisciplinary project [that] aims to investigate historical aspects of the arrival and spread of the disease in Latin America and, to make comparative studies of the history of the disease on both [the African and South American] continents" [76]. The lack of any follow up papers reporting substantial results suggests that this incipient idea was not further developed.

### Dengue

Of the four areas of NTDs research examined here, the largest body of social sciences research identified in Scopus belonged to dengue. A number of the studies identified by Scopus, however, were discarded from this analysis because they failed even the most basic test of being characterised as social sciences. Around 25% of the studies were on the relationship of climate change and the spread of dengue, but did not develop any particular social sciences theme e.g., [60,77-87]. A further group of studies covered a diversity of subjects from the development of insecticide impregnated fabrics [88,89], drug development [90], and clinical case studies [91], to mathematical models of disease transmission [92-94]. The extent to which these latter studies actually developed a social theme is hard to gauge. In a handful of studies the information contained in Scopus was too vague to make a determination. This left 42 studies from the originally extracted 74; and even among these 42 there was some doubt in our minds about whether they were truly social sciences articles.

The research did not divide neatly into themes in any single area, such as "implementation research", or studies of "knowledge attitude and behaviour", but could encompass multiple theme [95]. Health education [96-100], and related community perceptions studies were also themes of the social science research in dengue literature [101,102]. A common theme across many of the papers was the inclusion of a spatio-temporal component and the the use of geographic information systems (GIS) [103-107].

## Discussion

The major limitation of this review, as in most, is in the range and source of papers explored. The data were restricted by a search of the literature on only four of the NTDs, using one, albeit substantial database, with one set of rules defining social and biomedical sciences. Furthermore, the focus was on journals and not books.

The choice of disease was arbitrary and one might try to argue that different choices would see substantially different patterns of results. With the exception of HIV which is admittedly not a NTD, it is difficult to bring to mind any infectious disease that has a substantial social sciences research presence. This is an empirical question, however, and worthy of further research. Furthermore, alternative databases with alternative operational definitions of the sciences could yield somewhat different results. It should be remembered that in the sensitivity analysis of the onchocerciasis literature, a subsequent hand search of the database revealed many more social sciences research articles than were identified using the Scopus operational definition. If a hand search were conducted with the other diseases, similar results may occur - providing a possible explanation for the almost total absence of social science research in visceral leishmaniasis. This too is an empirical question and may be worthy of further investigation. The lack of books and book chapters in the database will also reduce the apparent presence of social science research. It is worth bearing three points in mind, however:

1. If the existence of a research article cannot be readily found in a search of the worlds largest database of research articles, then the impact of that article will be necessarily limited [108]. A search using typical default options is going to be preferred, and the literature identified in this way is more likely to inform policy, practice, and future research. This may also explain why the social sciences are tending to favour journals more than they have in the past [42].

2. Even if the number of social research articles (including interdisciplinary articles) identified in a hand search doubled or even tripled the total number of social science articles, it remains a disturbingly small quantum of the total research.

3. A substantial number of those articles identified in the default search as social science research were not. This creates a "two steps forward, one step backwards" effect, further reinforcing the point above about the proportionately small contribution of pure and interdisciplinary social science research to NTDs research.

The concern of this article was with the nature of the research as much as it was with the quantum of research. Although alternative search strategies may improve the identified quantum of research, there is no evidence to suggest that the nature of the unidentified research is substantially different from the identified research. It would be particularly disturbing if the unidentified research was in fact different in approach or better in quality.

The vast majority of the research literature related to the four NTDs chikungunya, dengue, onchocerciasis and visceral leishmaniasis belongs to biomedical research; and, most of that research belongs to the discipline of "medicine." Aggregating the abstracted research across all the disciplines of the social sciences and comparing it to the total published research, it is clear that the social sciences are neither a credible nor an important part of the research agenda for these NTDs.

The direction (although not the magnitude) of our findings could hardly be considered surprising. One would readily anticipate that biomedical research would form the greatest quantum of work in the field that is specifically disease focussed. That the social sciences contribution is only 2.1%, however, is confronting evidence of how inconsequential the social sciences are considered to be for understanding and managing these NTDs. Unfortunately, even among that 2.1%, significant amounts of research that were abstracted as associated with the social sciences, clearly were not. For instance, 5 out of the total of 7 "social sciences" research papers in the visceral leishmaniasis literature, were clearly unrelated to the social sciences. In the dengue literature, over 40% of the 74 purportedly social sciences research papers could be excluded on the basis of the title and abstracts alone, with a number of the remaining papers appearing not, or barely, to be social science related.

Things deteriorate on further examination. The figure of 2.1% combined the "pure" social sciences research with the cross disciplinary social and biomedical sciences research. This of course is inflated, because the incorrectly identified social science papers have not been excluded. If one disaggregates the cross disciplinary research and the "pure" social sciences research, the contributions were around 1% and 1.1% of the research literature, respectively.

One might counter that the social scientists are more likely to publish book chapters and books that may not be counted in this process. This is true. However, the approach we have taken to investigating the extent of the literature is also the kind of approach that

We would argue that, for the reasons previously identified, the social sciences, in and of themselves, have important contributions to make to NTDs research. Nonetheless, putting this notion aside, we focus on the cross disciplinary research. It is through inter-disciplinary (and to a lesser extent multi-disciplinary) research that the dynamic interplay between the vector, the host, and the pathogen within a social, cultural, physical and biological context can be understood. This would appear to be central to understanding and managing NTDs. Notwithstanding the importance of understanding NTDs as a dynamic process, the cross-disciplinary research accounts for barely 1% of the literature.

Where cross disciplinary research does occur, much of it is programmatic evaluation of the kind common in the onchocercal literature (e.g., [69,73]) or fairly rudimentary risk factors research (e.g., [57,58]). Programmatic evaluation is important, but it is also likely to constitute "hand maiden" research designed to support (and never challenge) the curative activities of biomedical research. If one of the core ideas underpinning scientific research is that it should challenge and confront existing dogma^4^, then this kind of adjuvant research, though utilitarian, does not contribute to the advancement of science because it is intended to support the activities of a dominant paradigm_5_. This is not to say that programmatic research cannot be, or is not, important. Indeed we (DDR and PA) have argued elsewhere that implementation research is critical [26], but it should not simply be about the identification of bottlenecks to programme delivery, but about generating new science. Parker and Allen in this series [31] provide a detailed case-study and discussion of the dangers associated with social science research as adjuvant research for biomedicine.

Given the paucity of "pure" social science research into these four NTDs, one would hope that the little research that is being conducted would be exceptional. There is no doubt that there is some important research being published. Examples such as the analysis of the policies of Metropolitan France and their effect on chikungunya on the Reunion and Mayotte islands [37]; the study of onchocerchal related stigma [74]; and the hint of a comparative, historical study of onchocerciasis in Africa and Latin America [76]; all point to the kind of critical social science research that is investigator driven, and is likely to have a direct impact on our understanding diseases in question, and when brought to the attention of policy makers should effect disease management. This is not to say that other social sciences studies did not do this, but rather that many of them conformed more to a model of adjuvant research described above rather than the kind of critical, investigator driven research that can guide our understanding and management of the NTDs in their own right.

In juxtaposing biomedical research and social science research, there is no criticism here of biomedical researchers. They have engaged well in that very social process of advocating for resources to advance their own agendas and careers (see Allotey et al. in this series [109]). The NTDs advocacy has relied on (a) appeals to alleviate the suffering of societies' most neglected, and (b) the scientific promise of the ultimate cure. Most of us would have attended an international conference where the promise of a vaccine to prevent, or a drug to cure, disease *X *is dangled enticingly, "because there have been important breakthroughs recently, and with an investment of only..." Most recently we have even had the idea of a "vaccine against poverty" peddled in the international literature (see Allotey et al. in this issue for a more complete discussion [109]). It is not difficult to see why funders and research priority setters are attracted to such claims; which even if mendacious - are well intentioned.

With far less appeal, social science research never claims cure, but it may occasionally claim prevention, although rarely with the same strident fervour. The "ultimate cure" claim, of course, is predicated on our capacity to ignore the dynamic interplay between the vector, host, and pathogen that occurs within a social, cultural, physical and biological context. The world of the ultimate cure is a flat, and unsubtle world, that rarely exists outside the confines of the petri dish or the tightly controlled clinical trial [110]. The development of drug resistance [111], non-compliance [14], lack of access [112], cultural beliefs [113], and the structure of the health system are a few of the very real issues for NTDs, which do not concern flat world science. They are nonetheless *the *important reality for social scientists. Indeed, in one very recent paper on the appearance of ivermectin resistant onchocerciasis, the biomedical researchers response was a textbook, flat world call for the development of "new therapeutic targets and agents ... desperately needed to treat and cure this devastating disease" (p.1) [111].

Virchow claimed that politics was medicine writ large, and it is hard not to see the links between power processes and the choices that are made about how disease research is conceptualised, managed, prioritised, and funded. The choices then have a direct effect on the ways that we come to understand and manage NTDs. Flat world research is surely a simpler message for funders and research priority setters to digest than a research agenda that speaks to real world complexity and does not even pretend to promise an ultimate cure. Solutions for this less complex world are also easier for funders and priority setters to on-sell to their political masters. Thus, where there is no criticism of the biomedical sciences, there is a serious and pointed criticism to be made of those who fund research and those who set research priorities.

Social science research, and interdisciplinary research should *not *be funded because social scientists want a larger piece of the pie. It should be funded because human pathogenesis never occurs in a Petri dish, and it occurs only relatively rarely within the confines of the clinical trial. If we are to understand and manage human pathogenesis on the scale of populations, then there are important social and interdisciplinary questions to be addressed in NTDs research.

Although we suggest that social scientists are not doing as much NTDs research as they *should *we also recognise the realities of the research funding landscape. In a high profile article from the late 1990s, the epidemiologist Kenneth Rothman objected to the view that anyone would claim that poverty was a concern for epidemiologists [114]. The objection was not that, as people, epidemiologists should not be concerned with poverty. Rather, the objection was that poverty was not of concern to epidemiology as a scientific endeavour. Furthermore, he argued, epidemiologists should be free to select the areas of research that they think are most relevant or interesting. Perhaps social scientists hold similar views about the irrelevance of NTDs research, and this explains the small presence of the social sciences in the NTDs literature.

The reality is, however, that where the funders and research priority setters may not be able to force epidemiologists to study poverty, or social scientists to conduct NTDs research, they do have powerful and persuasive, policy and financial instruments at their disposal. These instruments could make almost anything interesting to most scientists, because the science has not only been identified as important, it has been identified as fundable. It is for this reason that the failure to generate more social science research and much more interdisciplinary research on NTDs lies squarely at the feet of funders and priority setters.

## Conclusion

Infectious diseases represent a rich and dynamic interplay between the vector (where applicable), the host, and the pathogen. The interaction is complex and evolves within a social and cultural context as much as it does within a physical and biological context. Understanding this complex dynamic is crucial for the sustainable management of the NTDs.

The evidence from the literature, however, is that there is little investigator driven social science to speak of in the NTDs, and a similarly poor presence of interdisciplinary science. Without this, our understanding and management of NTDs is inevitably reduced to a strategy that relies on a repetitive, reductionist, flat-world science to overcome an acknowledged complex system.

NTDs research needs more sophisticated funders and priority setters who are not beguiled by pharmaceutical fairy-tales. Pharmaceuticals (including vaccines) are critical, but they are not the only solutions, and their final application is in complex dynamic worlds in which bug (and vector) evolution exploits the social nature of humans, and our responses have counter responses.

The current understanding of the dynamic, and the understanding of how to develop sustainable approaches to disease management are poor. There are no research templates to overcome this, and the silos of current NTDs science has discouraged the development of genuinely interdisciplinary research.

As a major recommendation there is a need to reconceptualise the outcomes for addressing the vulnerability of those who get NTDs, and the need to reconceptualise the ways in which the health needs of the neglected, poor, disenfranchised and dispossessed are managed. Recognising that the challenges cannot be reduced to solutions that exist outside a real world context is a first step.

## Competing interests

The authors declare that they have no competing interests.

## Authors' contributions

DDR, PA, and SP conceived the study jointly. DDR conducted the research and wrote the first draft. DDR, PA, and SP edited subsequent drafts. All authors read and approved the final manuscript.

## Appendix

1. A quote from a biomedical scientist sitting on a panel reviewing health and medical research proposals. Homologues of this will be familiar to many social scientists working in global health.

2. We use the terms "inter-disciplinary," "multi-disciplinary," and "cross disciplinary" in three distinct ways. We use "inter-disciplinary" to refer to research that brings different disciplines together to obtain a synthesis of ideas that are unobtainable from any one discipline. We use "multi-disciplinary" to refer to research that brings together more than one discipline, but the disciplinary contributions are distinct; thus, within a single research programme microbiologists will focus on questions relevant to microbiology and economists will focus on questions relevant to economists. We use "cross disciplinary" to refer to research generically that is either inter- or multi-disciplinary.

3. See, for instance, the paper by Allotey, et al [109] in this collection.

4. "Falsification" in a Popperian nomenclature [115]

5. "Normal science" in a Kuhnian nomenclature [116]
